# Fish *Snx27* promotes viral products by modulating the innate immune response and exosomal machinery

**DOI:** 10.1128/jvi.00974-24

**Published:** 2024-11-04

**Authors:** Yepin Yu, Jiaxin Liu, Zhiwen Zhao, Xiaoming Lan, Linmiao Li, Junjie Hu, Ying-an Zang, Xiujuan Zhang, Jinping Chen

**Affiliations:** 1 Guangdong Key Laboratory of Animal Conservation and Resource Utilization, Institute of Zoology, Guangdong Academy of Sciences, Guangzhou, Guangdong, China; 2 Guangdong Provincial Key Laboratory of Aquatic Animal Disease Control and Healthy Culture, College of Fisheries, Guangdong Ocean University, Zhanjiang, Guangdong, China; 3 Biosafety Laboratory, Guangdong Second Provincial General Hospital, Jinan University, Guangzhou, Guangdong, China; 4 College of Animal Sciences and Technology, Zhongkai University of Agriculture and Engineering, Guangzhou, Guangdong, China; 5 School of Life Science, Guangzhou University, Guangzhou, Guangdong, China; University Medical Center Freiburg, Freiburg, Germany

**Keywords:** sorting nexin 27, nervous necrosis virus, exosomes, immune response, ALIX, grouper

## Abstract

Viral nervous necrosis caused by the nervous necrosis virus (NNV) poses a significant threat to the global aquaculture industry. Developing preventive methods to minimize economic losses due to NNV infections is crucial. This study explored the role of the sorting nexin 27 (*Snx27*) gene, encoded by the orange-spotted grouper (*Epinephelus coioides*) and referred to as *EcSnx27*, as an immune regulator affecting red-spotted grouper nervous necrosis virus (RGNNV) infection *in vitro*. Our findings revealed that *EcSnx27* negatively regulates interferon (IFN)-related cytokines and the promoter activities of fish ISRE and NF-κB. Furthermore, we identified the SNX-FERM and SNX-FERM-like domains as responsible for the interaction between *EcSnx27* and RGNNV coat protein. Through the detection of viable virions associated with *EcSnx27*-containing exosomes, we propose that *EcSnx27* may contribute to the release process of RGNNV by influencing the apoptosis-linked gene 2-interacting protein X (ALIX)-associated exosomal pathway. Consequently, our study suggests that *EcSnx27* promotes RGNNV replication by inhibiting the IFN immune response and facilitating virus production and release through ALIX-mediated exosomal machinery.

**IMPORTANCE:**

Red grouper nervous necrosis virus (RGNNV), a member of the *Nodaviridae* family, has emerged as a significant cause of fish diseases worldwide, leading to high morbidity and mortality rates. This study investigated the sorting nexin 27 (*Snx27*) gene encoded by the orange-spotted grouper (*Epinephelus coioides*) on RGNNV infection in grouper kidney cells. Our findings revealed that *EcSnx27* negatively regulated the interferon pathway, resulting in the promotion of RGNNV replication. Additionally, we observed that *EcSnx27* could interact with apoptosis-linked gene 2-interacting protein X (ALIX) and the RGNNV coat protein, suggesting its potential involvement in viral release processes through modulation of the exosomal pathway. Our study identified *EcSnx27* as a key target that RGNNV exploits to enhance viral production. This finding offers valuable insights into the immune evasion and viral release mechanisms of non-enveloped RNA viruses.

## INTRODUCTION

As a member of the family *Nodaviridae*, the nervous necrosis virus (NNV) is the causative agent of viral nervous necrosis (VNN) disease (1). The VNN disease has become a devastating problem threatening the aquaculture industry and causing global economic losses (2). The NNV can infect more than 120 fish species worldwide and cause the breakdown of the central nervous system leading to nearly 100% mortality, particularly in the larval and juvenile stages (3–5). Currently, this infectious disease is prevented and controlled using antibiotics, vaccines, and other drugs. However, little effect has been achieved, but antibiotic pollution and water contamination occurred (6). Therefore, understanding the molecular mechanisms underlying NNV infection and identifying functional antiviral genes for application in VNN disease control is vital.

Sorting nexins (SNXs) are conserved proteins that localize to the cytoplasm and are related to organelle motility, cell signaling, protein sorting, and membrane remodeling/trafficking (7, 8). Constituted by the SNXs and vacuolar protein-sorting subcomplex, the retromer components undergo retrograde transport from endosomes to the trans-Golgi network or plasma membrane (7, 9, 10). The specific phox homology (SNX-PX) domain involved in phosphatidylinositol binding symbolizes SNX family members (11). The SNX-FERM subfamily, encompassing three members: SNX17, SNX27, and SNX31, is defined by the presence of an SNX-FERM domain that interacts with the NPxY/NxxY motifs of many transmembrane proteins (12, 13). Furthermore, with its unique SNX-PDZ domain, *Snx27* regulates the endosomal trafficking of PDZ-binding motifs containing cargoes, including the β2 adrenergic receptor and glucose transporter GLUT1 (14–16).

Exosomes are nanovesicles (30–150 nm in diameter) generated through the endosomal pathway. They are formed within multivesicular bodies as intraluminal vesicles (ILVs) (17). The SNXs are a family of proteins that play crucial roles in various cellular processes, including the formation and trafficking of intracellular vesicles. Certain SNXs have been associated with this process in the context of exosome formation. SNX2 has been implicated in ALIX (ALG-2 [apoptosis-linked gene 2]-interacting protein X)-mediated exosomal function (18), and the SNX31 mediates the formation of ILVs and exosomes (19). Alternatively, several studies have documented the significant roles of exosomes in intercellular communication in several physiological and pathological processes and the spread of viruses (20–22). It has been revealed that the Epstein–Barr virus (EBV) modulates the CD63 interactome through viral latent membrane protein 1, and the SNXs have been identified to interact with CD63 (23, 24). Hence, SNXs could play a role in virus infection and release through modulation of the exosomal machinery.

Other than being the retromer component, SNX family members participate in the cell signaling transduction related to immune regulation, inflammatory modulation, and antiviral response (25, 26). It has been demonstrated that *Snx27* inhibits tumor necrosis factor alpha (TNFα)-induced NF-κB signaling activation by regulating the TNFα receptor complex associated with ovarian tumor (OTU) deubiquitinase with linear linkage specificity (OTULIN) and linear ubiquitination (27, 28). Besides, SNX5 impairs RIG-I-like receptor (RLR)-mediated antiviral signaling (29), whereas SNX8 functions as an anti-bacterial factor by mediating the interferon (IFN)-triggered noncanonical signaling pathway (30). However, whether and how *Snx27* is involved in IFN-related cytokine modulation remains unclear. In this study, we explained the underlying mechanisms of fish encoding the *Snx27* gene in immune regulation and viral infection.

This study aimed to characterize the *Snx27* gene in orange-spotted grouper (*Epinephelus coioides*) and elucidate its role as a regulator of the immune response related to IFN. Overexpression of this orange-spotted grouper sorting nexin 27 (*EcSnx27*) in grouper kidney (GK) cells resulted in the downregulation of immune cytokine expression and reduced activity of the interferon-stimulated response element (ISRE) and NF-κB promoters. Consequently, increased *EcSnx27* expression promoted the replication of RGNNV, whereas *EcSnx27* knockdown by short hairpin RNA (shRNA) significantly inhibited viral replication. Moreover, the involvement of fish *Snx27* in viral transport and release processes through the ALIX-mediated exosomal pathway was demonstrated by the interaction of *EcSnx27* and ALIX and the colocalization of *EcSnx27* with the coat protein (CP) of RGNNV, as well as the infectious exosomes associated with *EcSnx27* isolated from infected GK cell culture medium. These findings confirmed the contribution of fish *Snx27* in facilitating viral dissemination and release through exosomal machinery.

## MATERIALS AND METHODS

### Culturing cell lines and RGNNV replication

Epithelial-like cells developed from the kidney tissue of the grouper (*E. coioides*) were named GK cells. The GK cells were cultured in Leibovitz’s L-15 medium (Life Technologies, USA) supplemented with 10% heat-inactivated fetal bovine serum (FBS, Gibco, USA) at 28°C. Cells were passaged every 3 days in 25-cm^2^ flasks (ThermoFisher, USA). Red-spotted grouper nervous necrosis virus (RGNNV) was propagated and cultured in GK cells. Viral-infected cells were freeze–thawed thrice to release the virus particles and stored at −80°C until further use.

293T cells were from ATCC. Cells were cultured with Dulbecco’s modified Eagle’s medium (Corning, USA) containing 10% FBS and 1% penicillin/streptomycin (Gibco, USA) at 37 C in 5% CO_2_ incubator.

### Cloning and sequence analysis of the *EcSnx27*

Referring to the transcriptome data (31) and the GenBank database, primers used for the full-length amplification and sequencing of *EcSnx27* were designed with Primer Premier 5 (http://www.premierbiosoft.com/; Table S1). A polymerase chain reaction (PCR) assay was employed to obtain the open reading frame (ORF) of the *EcSnx27* gene using the *EcSnx27*-seq-PF/PR (Table S1). Total RNA isolated from GK cells was reverse transcribed into cDNA using the PrimeScript RT reagent Kit (Takara, Japan). The PCR reaction was then performed in a 50-µL system containing 25 µL of 2× Mix (Genesand, China), 40 ng of cDNA templates, forward and reverse primers (0.2 µM each), and ddH_2_O (up to 50 µL). DNA sequencing was subsequently performed for the amplicons from Sangon Biotech.

### Constructing plasmids for gene overexpression and knock-down

The Uniclone One Step Seamless Cloning Kit (Genesand, China) was used to construct the certain sequences into vectors N_3_-GFP or pSilencer-4.1-CMV-neo. Suitable restriction enzymes were used for empty vector linearization. *XhoI* and *KpnI* were used to digest the N_3_-GFP vector, whereas *BamHI* and *HindIII* were used for pSilencer-4.1-CMV-neo vector linearization.

To achieve gene overexpression, the ORF of *EcSnx27* was cloned into an N_3_-GFP vector following the manufacturer’s protocol. First, PCR amplification was performed using primers *EcSnx27*-GFP-PF/PR (Table S1). Domain-deleted fragments were amplified using the overlapping PCR method as described previously (32). The recombinant vector was constructed in the following reaction system: 5 µL of 2× Uniclone Seamless Cloning Mix (Genesand), 1 µL of linearized empty vector, and 4 µL of PCR products. The reaction was processed at 50°C for 5–15 min, followed by transformation.

For *EcSnx27* knockdown, two shRNAs (*EcSnx27*-shRNA1 and *EcSnx27*-shRNA2, Table S2) were designed using the online shRNA designer (https://rnaidesigner.thermofisher.com). Similar steps were conducted to clone shRNAs into the pSilencer-4.1-CMV-neo plasmid. The recombinant vectors were verified by restriction enzyme digestion and DNA sequencing as described. To achieve the knockdown of ALIX, small interfering RNAs (siRNAs) were designed and synthesized by Genecreate Biological Engineering Co., Ltd. (refer to Table S2). GK cells were transfected with the siRNAs, with a negative control (nc) utilized as the internal control. The efficiency of the knockdown was assessed through western blotting.

The 3 HA*-Cp* plasmid was constructed using the primers listed in Table S1 and was used as a template to obtain the standard curve of the absolute quantitative PCR (qPCR). The plasmid was also used in co-immunoprecipitation (Co-IP) assay for the viral CP expression purpose.

### Transfecting plasmids and selecting stable cell lines

Lipofectamine 2000 reagent (Invitrogen, USA) was used for cell transfection, following the manufacturer’s instructions. Briefly, GK cells were seeded in a 24-well plate and grown to 70%–80% confluence for transfection. The culture medium was then replaced with an FBS free L15 medium containing the mixture of plasmids and Lipofectamine 2000 reagent. After incubation for 6 h, cells were cultured in an L15 medium supplemented with 10% FBS for further analysis.

The G418 (Gibco, USA) was used in stable cell line selection. Cells were trypsinized and generated with a complete medium containing 10% FBS and G418 (800 ng/mL) after 48 h post transfection. Cells were propagated under the selective pressure for several weeks, and stable cell lines were confirmed using quantitative real-time PCR (qRT-PCR) and western blot analysis.

### Isolating the total RNA and qRT-PCR analysis

The total RNA of GK cells was isolated using a TRIzol reagent (Life Technologies, USA). To examine the expression pattern of *EcSnx27* after NNV infection, GK cells were seeded in a 24-well plate before virus infection at a multiplicity of infection (moi) of 0.5. Cells were harvested at the indicated time points (0, 6, 12, 24, 36, and 48 h) post viral infection followed by RNA isolation. To detect overexpression or knockdown of the *EcSnx27* gene affecting viral infection, transfected or stable cells were infected with RGNNV at an moi of 1. Cells were harvested at 24 and 48 h p.i. (post infection). Total RNA was extracted, and 1  µg of cDNA was subsequently synthesized using the SuperScript III First-Strand Synthesis System (Invitrogen, USA) for further qRT-PCR analysis.

The qRT-PCR was performed with a 20-µL reaction system containing 10 µL of 2× qPCR Master Mix (Takara), 500 nM of each primer (Table S1), 1 µL of 5× diluted template cDNA, and ddH_2_O (up to 20 µL). The expression levels of each target gene were normalized with the 2^−ΔΔCT^ method (33) using the housekeeping gene elongation factor 1-alpha 1 (*Ef1α*) as the internal control. The results are represented as means ± standard deviation (SD), and the statistical significances were determined with a Student’s *t*-test and established at *P* < 0.05 (*).

### Dual-luciferase reporter assay

To demonstrate the regulatory effect of *EcSnx27* on the interferon promoter activity, reporter plasmids, including ISRE-Luc or NF-κB-Luc, were co-transfected with the internal control plasmid *Renilla* luciferase and N_3_-GFP or N_3_-*EcSnx27* in GK cells, respectively. After 48 h post-transfection, cells were collected with a lysis buffer. The dual-luciferase reporter assay was performed using the Dual-Luciferase Reporter Assay System (Vazyme, China), following the manufacturer’s instructions. The results were represented as means ± SD, and the statistical significances were determined with a Student’s *t*-test and established at *P* < 0.05 (*).

### Fluorescence microscopy

As described previously, GK cells seeded in glass-bottom culture dishes (Biosharp, China) were transfected with N_3_-GFP or N_3_-*EcSnx27* plasmids. At 48 h post transfection, cells were fixed with 4% paraformaldehyde and stained with Hoechst 33342 (Sigma-Aldrich, USA). Green signals representing the localization of green fluorescent proteins were observed under a fluorescence microscope.

To determine the colocalization between *EcSnx27* and RGNNV-CP, GK cells were transfected with N_3_-GFP or N_3_-*EcSnx27* plasmids, respectively. Cells were infected with RGNNV (at an moi of 1.0) for 24 h and fixed with 4% paraformaldehyde. An immunofluorescence assay (IFA) was performed using the anti-RGNNV-CP antibody as previously described (4). Cells were treated with 0.25% Triton X-100 (Sigma-Aldrich, USA) in phosphate-buffered saline (PBS) and blocked with 5% bovine serum albumin (BSA) in PBS before incubation with anti-RGNNV-CP antibody (1:200) at 4 ˚C overnight. The cells were washed thrice with PBS, incubated with Alexa Fluor 568 dye anti-rabbit antibody (ThermoFisher, USA), stained with Hoechst 33342, and observed under an FV3000 confocal microscope (Olympus Corporation, Japan).

GK cell co-transfection was carried out using 3 HA*-Cp* plasmid together with N_3_-*EcSnx27*, N_3_-∆PDZ, N_3_-∆PX, N_3_-∆FERM, or N_3_-∆FERM-like at a 1:1 ratio, respectively. Cells were fixed 48 h post-transfection. After treated with Triton X100, cells were blocked using 2% BSA. Then, the anti-HA antibody (Cell signaling, Cat No.: 2367) and Goat Anti-Mouse IgG H&L (Abcam, Cat No.: ab150116) were used as the first and second antibody, respectively. After that, cells were stained using Heochst33342 and imaged by EVOS FL Auto (Life technologies).

### Virus titer assay

To uncover the effects of *EcSnx27* gene on viral production *in vitro*, GK cells expressing N_3_-GFP, N_3_-*EcSnx27*, pSilencer-Scramble, or pSilencer-*EcSnx27*-shRNA1 were infected with RGNNV (at an moi of 1). Cells and culture media were harvested at 24 and 48 h p.i.. After freeze–thawing thrice, the samples were centrifuged at 2,000 × *g* for 10 min. After serial 10× dilution, the supernatant-infected GK cells were seeded in a 96-well plate. The cytopathic effect (CPE) caused by virus infection wasobserved daily under a light microscope. The virus titers were measured with the 50% tissue culture infectious dose (TCID_50_) assay as described previously (34).

### Western blotting and co-immunoprecipitation (Co-IP) assay

Western blotting was performed to determine the impact of *EcSnx27* on viral replication. Briefly, cells expressing N_3_-GFP or N_3_-*EcSnx27* were infected with RGNNV for 24 and 48 h and harvested using Pierce IP Lysis Buffer (ThermoFisher, USA). After boiling with loading buffer for 10 min, the samples were separated by 10% sodium dodecyl sulfate polyacrylamide (SDS-PAGE) gel electrophoresis and transferred to 0.2-µm polyvinylidene difluoride membrane (PVDF membrane, Millipore, USA). The membranes were blocked with 5% skim milk and then incubated with anti-RGNNV-CP (1:1,000), anti-GFP (1:1,000, Proteintech, China, Cat No.: 50430–2-AP), anti-IRF3 (1:1,000, Proteintech, China, Cat No.: 11312–1-AP), anti-IRF7 (1:1,000, Proteintech, China, Cat No.: 22392–1-AP), anti-NF-κB (1:1,000, Proteintech, China, Cat No.: 10745–1-AP), anti-SNX27 (1:500, Proteintech, China, Cat No.: 68386–1-Ig), anti-CD63 (1:500, Proteintech, China, Cat No. 25682–1-AP), anti-ALIX (1:500, Proteintech, China, Cat No. 12422–1-AP), anti-Nedd4 (1:500, Proteintech, China, Cat No.: 21698–1-AP), anti-TSG101 (1:500, Proteintech, China, Cat No.: 28283–1-AP), or anti-*β*-tubulin (1:2,000, Proteintech, China, Cat No.: 10094–1-AP) overnight. Subsequently, the membranes were washed with PBST thrice and incubated with the secondary antibody (horseradish peroxidase-conjugated goat anti-mouse IgG [1:3,000, Abcam, USA, Cat No.: ab19195] or goat anti-rabbit IgG [1:3,000, Abcam, USA, Cat No.: ab6721]) for 1 h. After washing with PBST, specific binding was observed using Pierce ECL western blotting Substrate (ThermoFisher, USA). The mean gray value of each bind was calculated with Image J (https://imagej.en.softonic.com/), and the data were normalized to the mean of *β*-tubulin expression. The results are representative of three independent experiments.

For the Co-IP assay, GK cells were seeded into 10-cm dishes (Corning, USA) overnight and transfected with N_3_-GFP or N_3_-*EcSnx27* plasmid, respectively. After 24 h post transfection, cells were infected with RGNNV (at moi of 5.0), washed with ice-cold PBS buffer, and lysed with IP lysis buffer (ThermoFisher, USA) supplemented with 100 µg/mL of RNase. After centrifugation (12,000 × *g*, 3 min at 4°C), the supernatants were collected for the IP assay using the Dynabeads Protein G Immunoprecipitation Kit (ThermoFisher, USA) following the protocol. Dynabeads Protein G was incubated with an anti-GFP antibody (1:200) for 20 min on ice and then incubated with the supernatant containing the antigen (Ag) at 4°C overnight. The Dynabeads–Ab–Ag complex was washed with washing buffer and resuspended in 20 µL of elution buffer. Anti-enhanced green fluorescent protein (EGFP), anti-RGNNV-CP, and anti-*β*-tubulin antibodies were used for immunoblotting.

293T cells seeded in the 10-cm dishes (Corning, USA) were co-transfected with the plasmid set of N_3_-GFP and 3 HA-CP, or N_3_-*EcSnx27*3 and 3 HA-CP, respectively. Cells were collected after 48 h post transfection. IP assay was performed using anti-GFP antibody following the manufacturer’s instructions. The whole cell lysate and IP products were analyzed by western blotting using anti-GFP, anti-HA, and anti-*β*-tubulin antibodies.

To determine the exosome-related protein that interacts with *EcSnx27*, GK cells seeded in 10-cm dishes (Corning, USA) were collected with IP lysis buffer. The IP assay was performed with anti-ALIX, anti-CD63, or anti-TSG101 antibodies. The IgG was used as the control. Immunoblotting analysis was subsequently conducted with the indicated antibodies.

### Exosome isolation

GK cells were seeded in 10-cm dishes (Corning, USA) overnight, followed by mock or RGNNV infection. After incubation with the virus for 6 h, the cells were washed by PBS buffer three times, and then cultured in Leibovitz’s L-15 medium (Life Technologies, USA) supplemented with 10% exosome-depleted fetal bovine serum (Bide, China). The exosomes were isolated using the Total Exosome Isolation Reagent (from cell culture media, ThermoFisher, Cat No.: 4478359) following the manufacturer’s instructions. Briefly, the supernatant was collected and centrifuged (2,000 × *g* for 30 min). The supernatant containing cell-free culture medium was transferred to a new tube without disturbing the pellet. Then, 0.5 volumes of the total exosome isolation (from cell culture media) reagent (Invitrogen, USA) were added and mixed well by pipetting up and down. The samples were incubated at 4 ˚C overnight. After incubation, samples were centrifuged at 10,000 × *g* for 1 h at 4 ˚C. The supernatant was discarded, and exosomes were contained in the pellet at the bottom of the tube. The pellet was resuspended in a convenient volume of 1× PBS for downstream analysis.

To avoid the influence of *Snx27* on virus entry (35), we performed RGNNV infection before N_3_-GFP and N_3_-*EcSnx27* transfection. Briefly, GK cells were seeded in 10-cm dishes (Corning, USA) overnight. Cells were infected with RGNNV (at an moi of 2.0) and incubated at 4 ˚C for no more than 30 min. After the virus adhered to the cell membrane, cells were washed with PBS thrice and cultured with the complete culture medium (supplemented with 10% exosome depleted FBS) for another 30 min at 28 ˚C to trigger virus internalization. Cell transfection was subsequently performed. The exosomes were isolated as described previously, and the total protein contained in the extracted exosomes were quantified using the bicinchoninic acid (BCA) Protein Assay Kit (Sangon Biotech, Cat No.: C503021). Then, the samples were used to detect the impact of *EcSnx27* on the virus products within the exosomes.

### Cell treatment

Exosomes were isolated from the supernatants of RGNNV-infected GK cells treated with or without the nSMase2 inhibitor GW4869 (Sigma-Aldrich) at different dosages (2, 5, or 10 µM) as described previously (36). Briefly, different concentrations of GW4869 were added for cell incubation, and the same volume of dimethyl sulfoxide was used as the control. After 2 h of incubation, RGNNV at an moi of 1.0 was added. The cultures were removed at 6 h p.i., and the cells were washed thrice with PBS, and treated with the same doses of GW4869 in fresh L-15 supplemented with 10% exosome-depleted FBS to exclude residual virions during the initial infection. Exosomes were extracted using a previously described method at 24 h p.i. and stored at −80 ˚C for further analysis.

### Viral detection of the exosomes

Western blotting and PCR assays were conducted to detect virions in the extracted exosomes. As described above, the isolated exosomes resuspended in PBS were boiled in a loading buffer for 10 min before performing SDS-PAGE and western blot analysis. Regarding the viral RNA analysis, the QIAamp Viral RNA Mini Kit (Qiagen, Germany) was first used for viral RNA extraction following the manufacturer’s instructions. Then, cDNA was synthesized as the template accordingly and then used for qRT-PCR, absolute qPCR, or reverse-transcription PCR (RT-PCR) analysis. The primers RGNNV-Cp-PF/PR were used for the qRT-PCR and absolute qPCR assay (Table S1). The RT-PCR assay was performed with primers RGNNV-RNA1-I-PF/R, RGNNV-RNA1-II-PF/R, RGNNV-RNA1-III-PF/R, or RGNNV-RNA2-IV-PF/R (Table S1).

### Nanoparticle tracking analysis (NTA)

Isolated exosomes were examined with the NanoSight NS300 system for numbers and size distribution, and the data were analyzed using OriginPro (https://www.originlab.com/).

### *In silico* analysis

The 3D structures of RGNNV-CP and *EcSnx27* were predicted and illustrated using PyMol (Schrödinger, LLC; Version 1.2r3pre) and Alpha Fold (37). The HDOCK webserver was used to study protein–protein interactions (38). Based on the least energy score, the optimal binding conformations for RGNNV-CP and *EcSnx27* were selected from the docking results. Visual analysis was performed using PyMOL.

### Statistical analysis

The data are expressed as the mean ± SD. The significance of the variability between the different treatment groups was calculated with the two-tailed, unpaired Student *t*-test using GraphPad Prism 6.0 software (Graph Pad Software Inc.). A *P* value of < 0.05 was considered to indicate statistical significance.

## RESULTS

### *Snx27* is conserved in vertebrates

The ORF of the orange-spotted grouper (*Epinephelus coioides*) *Snx27* gene was 1,698 bp and encoded a polypeptide with 565 amino acids and weighted ~63.997 kDa. Sequence alignment analysis showed that the *Snx27* coding sequence in orange-spotted grouper shared 99.65% and 98.41% identity with that in giant grouper (*Epinephelus lanceolatus*) and yellow perch (*Perca flavescens*), respectively (Table S3), whereas it only shared 84.36% and 86.27% identity with human (*Homo sapiens*)- and zebrafish (*Danio rerio*)-encoded SNX27, respectively (Table S3). We also compared the similarities of each functional domain, and the results suggested that PX and FERM-like domains were comparatively more conserved (Table S3). As a result, the *Snx27* function should be conserved in vertebrates.

### *EcSnx27* is located in the cytoplasm

**Fig 1:**
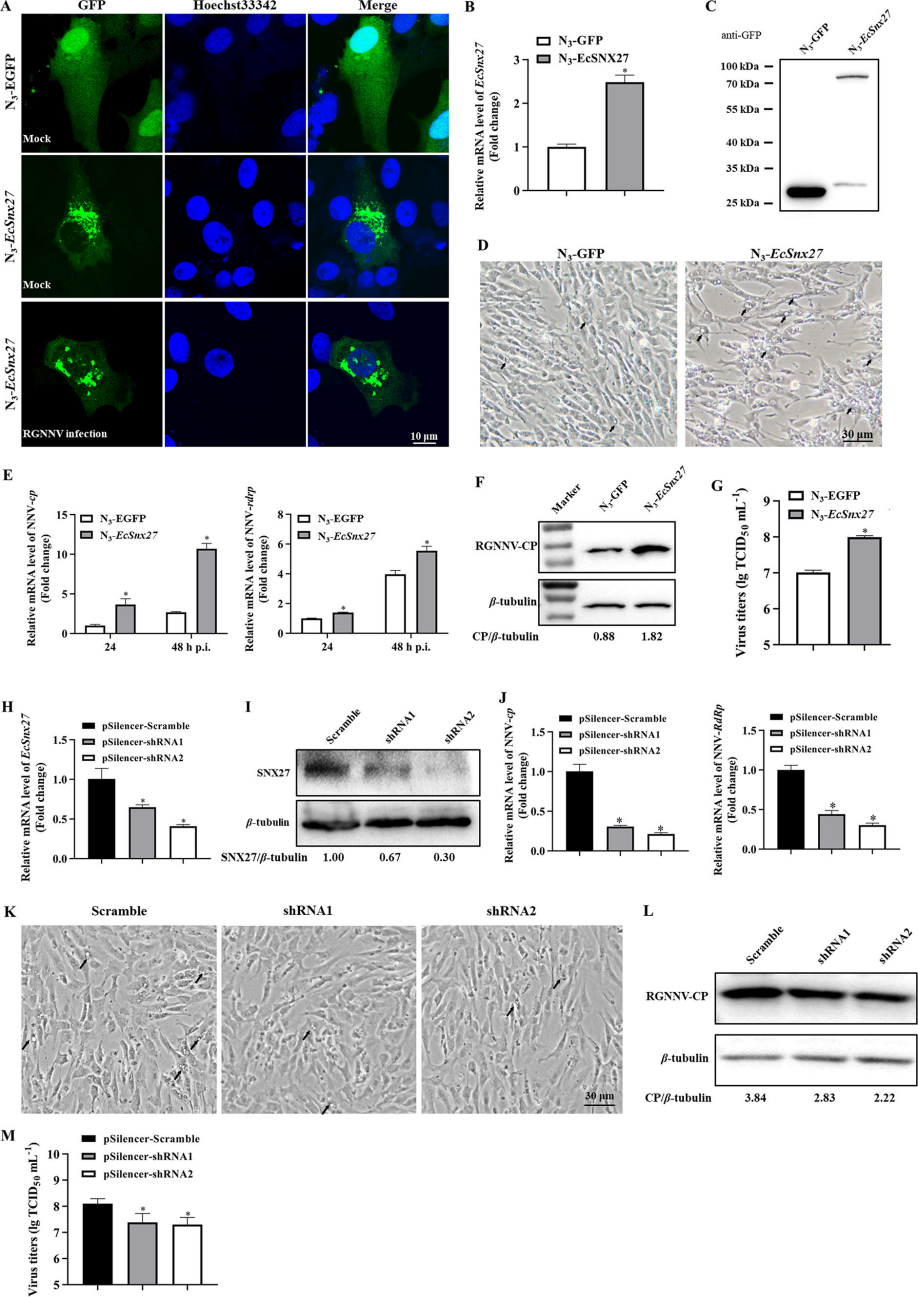
(**A**) Subcellular localization of the N_3_-EGFP (upper row), *EcSnx27*–EGFP fusion protein in GK cells (middle row), and the EcSNX27–EGFP fusion protein after RGNNV infection (bottom row). Overexpression of the *EcSnx27* gene was detected using qRT-PCR (**B**) and western blot assay (**C**). (**D**) *EcSnx27* facilitates the RGNNV-induced CPEs in GK cells. GK cells were transfected with the indicated plasmids and infected with RGNNV at an moi of 1.0. The CPE caused by RGNNV infection are indicated by the black arrows. Overexpression of the *EcSnx27* gene increased the transcriptional levels of RGNNV-*cp* and *rdrp*, as assessed by qRT-PCR (**E**), the protein level of RGNNV-CP detected by the western blotting (**F**), as well as the virus titers evidenced by the TCID_50_ assay (**G**). The knockdown efficiency of shRNAs were detected with qRT-PCR assay (**H**) and western blot analysis (**I**). (**K**) *EcSnx27* knockdown inhibited RGNNV-induced CPE in GK cells. Knockdown of the *EcSnx27* gene represses the transcriptional levels of RGNNV-*cp* and *rdrp* (**J**), the translational level of RGNNV-CP (**L**), and the virus titers (**M**). All data are presented as mean ± SD, *n* = 3. The significance of the variability between the different treatment groups was calculated with the two-tailed, unpaired Student *t*-test, *P < 0.05.

GKs were transfected with N_3_-GFP or N_3_-*EcSnx27* vectors for subcellular localization. Green fluorescence indicated the intracellular location of *EcSnx27*. Unlike control cells transfected with the N_3_-GFP vector, the GFP signals were observed throughout the cells (Fig. 1A, upper row); the *EcSnx27*-GFP fusion protein was predominantly distributed in the cytoplasm with some punctate fluorescence (Fig. 1A, middle row). The distribution pattern of *EcSnx27* was altered after RGNNV infection (Fig. 1A, lower row), exhibiting an increased punctate appearance and entering into the nuclear.

### *EcSnx27* facilitates RGNNV replication

*EcSnx27* overexpression was detected by qRT-PCR (Fig. 1B) and western blotting (Fig. 1C). The effects of *EcSnx27* on RGNNV infection were evaluated based on CPE progression, viral gene transcription, translation levels, and virus titers. The RGNNV-induced CPE was more pronounced in *EcSnx27*-expressing cells than in the control group (Fig. 1D). In *EcSnx27* overexpression cells, the transcription levels of RGNNV-*Cp* and RGNNV-*RdRp* genes and the translation levels of structural proteins of the virus were significantly elevated (Fig. 1E and F). Moreover, *EcSnx27* also increased viral production, as evidenced by a higher virus titer (Fig. 1G).

Conversely, the *EcSnx27* gene was knocked down by shRNA method and detected with qRT-PCR (Fig. 1H) and western blotting (Fig. 1I). *EcSnx27* knockdown repressed RGNNV infection, as evidenced by lower viral gene transcription levels (Fig. 1J), decreased virus-induced vacuoles observed (Fig. 1K), inhibited viral protein levels (Fig. 1L), and repressed virus titers (Fig. 1M). Accordingly, the *EcSnx27* gene might benefit the RGNNV infection in fish cells.

### *EcSnx27* affects immune response *in vitro*

**Fig 2:**
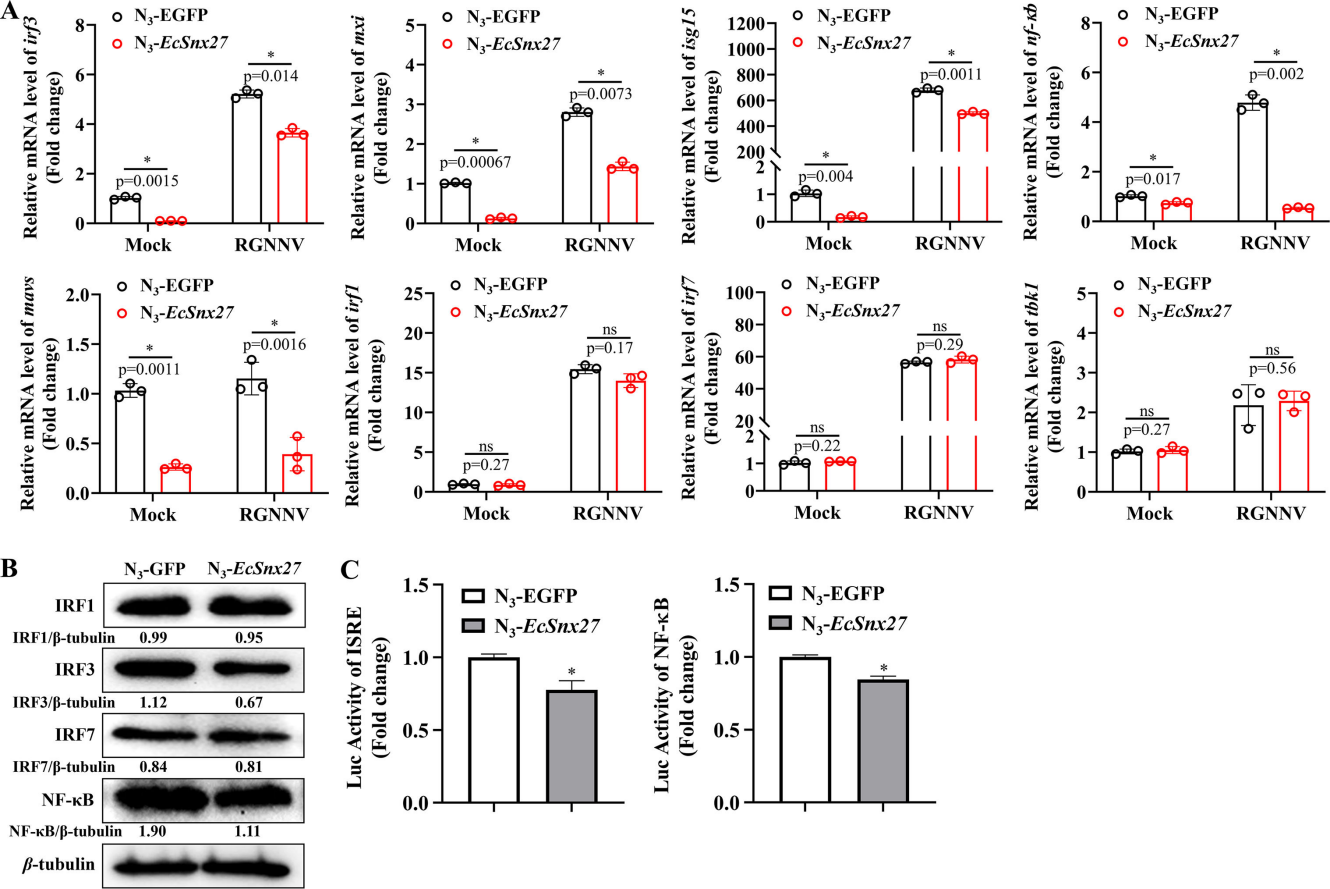
*EcSnx27* negatively regulates IFN response in GK cells. (**A**) The transcriptional levels of IFN-associated genes after *EcSnx27* overexpression were evaluated with qRT-PCR. Fish *Irf3*, *Isg15*, *MxI*, *Nf-κb,* and *Mavs* were downregulated by *EcSnx27* with or without RGNNV infection, while *EcSnx27* has no effects on modulating the mRNA level of *Irf1*, *Irf7*, and *Tbk1* gene. (**B**) Similar to the transcription regulating manner, *EcSnx27* decreases the protein levels of the *Irf3* and *Nf-κb*. But *EcSnx27* has no significant impacts on *Irf1* or *Irf7* expression levels. The numbers referring to the ratio of the indicated proteins and the *β*-tubulin is shown. (**C**) *EcSnx27* decreases the promoter activities of ISRE and NF-κB. GK cells were co-transfected with ISRE-Luc/NF-κB-Luc, *Renilla* luciferase vector, and N_3_-GFP or N_3_-*EcSnx27*. The promoter activities were measured using the luciferase reporter gene assay. Setting the promoter activities of the N_3_-GFP group as onefold. All data are presented as mean ± SD, *n* = 3. The significance of the variability between the different treatment groups was calculated with the two-tailed, unpaired Student *t*-test, *P < 0.05; ns, no significance.

To investigate the potential role of *EcSnx27* in the immune response, we measured the mRNA expression levels of IFN-related molecules after *EcSnx27* overexpression. RGNNV infection significantly induced the mRNA levels of all the detected immune genes. Interestingly, *EcSnx27* downregulated the interferon regulatory factor 3 (*Irf3*), IFN-stimulated gene 15 (*Isg15*), MX dynamin-like GTPase 1 (*Mx1*), nuclear factor-kappa B (*Nf-κb*), and mitochondrial antiviral signaling protein (*Mavs*) gene significantly in both mock- and RGNNV-infected cells (Fig. 2A). Moreover, *EcSnx27* modulated IFN-related molecules by repressing *Mavs* and *Irf3*, without affecting the expression of *Irf1*, *Irf7*, or TANK-binding kinase 1 (*Tbk1*) (Fig. 2A). Furthermore, *EcSnx27* significantly inhibited the protein levels of *Irf3* and *Nf-κb* without affecting *Irf1* or *Irf7*, as shown in the western blotting assay (Fig. 2B). Consistently, the promoter activities of ISRE and NF-κB were inhibited by *EcSnx27*, as suggested by the dual-luciferase reporter assay (Fig. 2C). Thus, we proposed that *EcSnx27* could negatively regulate fish cells’ IFN response signal pathway.

### SNX-FERM domains are responsible for the interaction between *EcSnx27* and RGNNV-CP

**Fig 3:**
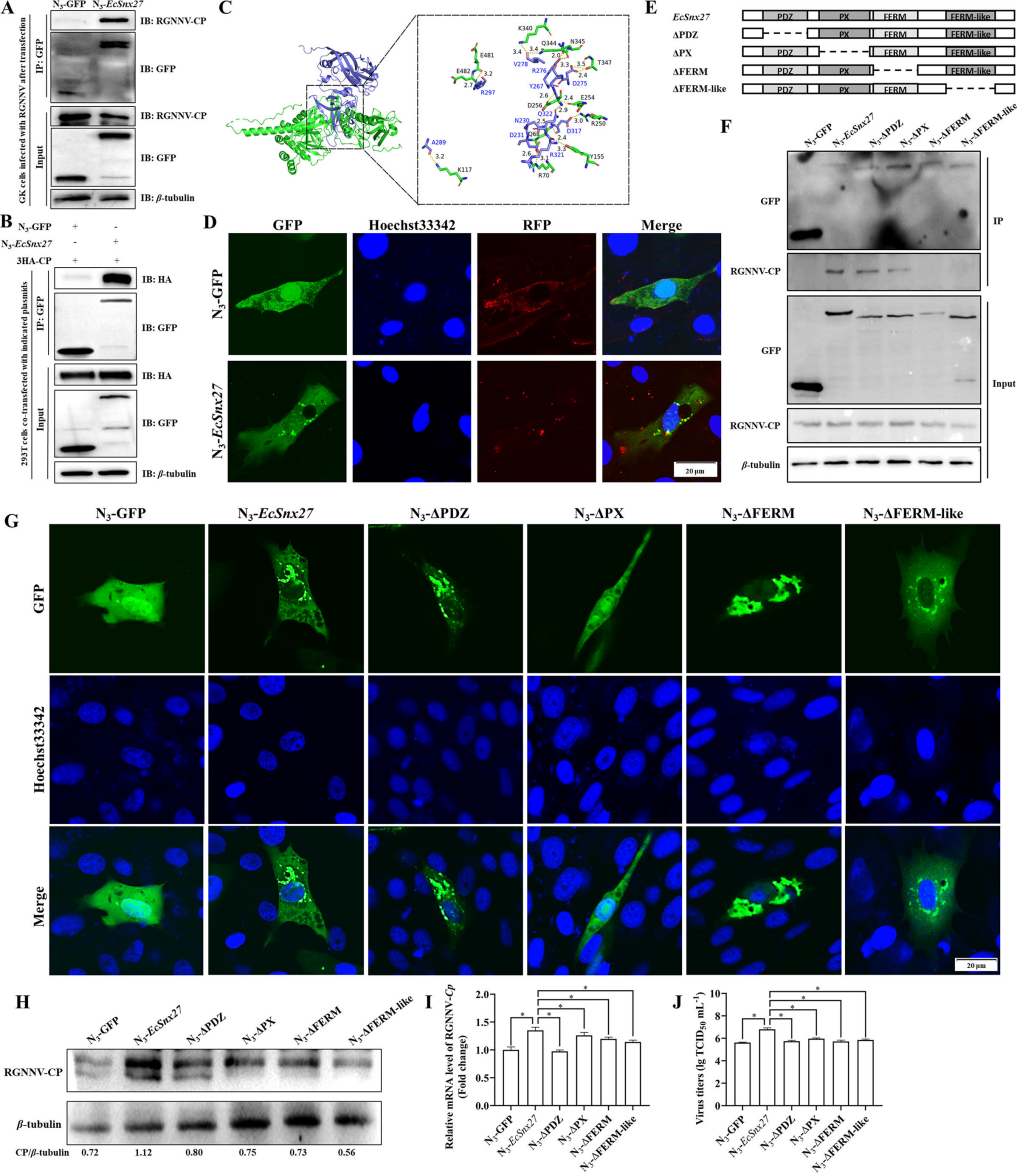
*EcSnx27* interacts with RGNNV-CP. (**A**) The Co-IP assay revealed the interaction between *EcSnx27* and RGNNV-CP. GK cells were transfected with N_3_-GFP or N_3_-*EcSnx27* for 24 h, and subsequently infected with RGNNV at an moi of 5.0. The cells were lysed and immunoprecipitated with the Dynabeads Protein G incubated with anti-GFP antibody. Then, immunoblotting assay was performed using anti-GFP, anti-*β*-tubulin, and anti-RGNNV-CP antibodies, respectively. (**B**) 293T cells seeded in 10-cm dishes were co-transfected with indicated plasmids (N_3_-GFP and 3 HA-CP or N_3_-*EcSnx27* and 3 HA-CP), respectively. Co-IP assay was performed using anti-GFP antibody followed by immunoblotting analysis. The interaction between RGNNV-CP and *EcSnx27* was confirmed. (**C**) Crystal structure of RGNNV-CP/*EcSnx27* complex. Left: *EcSnx27* (green) bound to RGNNV-CP peptide (sky blue). Right: Detailed interactions of the *EcSnx27* (green) bound to RGNNV-CP peptide (sky blue). The residues involved are labeled. H bonds are shown in dashed line. (**D**) Co-localization of the *EcSnx27* and RGNNV-CP was evaluated by IFA and observed under a confocal microscope (Olympus). GK cells were transfected with N_3_-EGFP or N_3_-*EcSnx27,* respectively. After RGNNV (at an moi of 2.0) infection for 24 h, cells were fixed, and cell immunofluorescence staining was performed using anti-RGNNV-CP antibody. Cell nuclei were stained with Hoechst33342. (**E**) Diagram of *EcSnx27* and its domain-deleted mutants ΔPDZ, ΔPX, ΔFERM, and ΔFERM-like. (**F**) *EcSnx27* interacts with RGNNV-CP via FERM and FERM-like domain documented by Co-IP assay. The plasmids of the empty control and the full-length or truncated *EcSnx27* were transfected into GK cells seeded in 10-cm dishes. After 24 h post transfection, cells were infected with RGNNV (at an moi of 5.0), and the cell lysate was used for the Co-IP assay using anti-GFP antibody followed by western blotting. (**G**) Analysis of the subcellular localization of *EcSnx27* and the indicated mutations were performed. GK cells were fixed after 48 h post transfection. The nucleus was stained with Hoechst33342, and the images were taken with the confocal microscope (Olympus). The PDZ, FERM or FERM-like domains had little impacts on the localization, whereas the SNX-PX domain was responsible for the subcellular localization of *EcSnx27*. (H to J) Western blot assay (**H**), qPCR analysis (**I**), and TCID_50_ assay (**J**) were carried out to evaluate the deletion of different domains on RGNNV replication. The data are presented as mean ± SD, *n* = 3. The significance of the variability between the different treatment groups was calculated with the two-tailed, unpaired Student *t*-test. An asterisk indicates a comparison with the N3-*EcSnx27* group. *P < 0.05.

Members of the SNX family can interact with several host and virus proteins, including CD63 (23), MITA (26), OTULIN (27), and the structural protein of the human respiratory syncytial virus (39). Our Co-IP assay showed that RGNNV-CP could be immunoprecipitated in *EcSnx27*-transfected cells, but not in control cells (Fig. 3A). A similar result was suggested by the co-transfection assay that there was an interaction between RGNNV-CP and *EcSnx27* (Fig. 3B). The molecular docking results demonstrated a relatively stable RGNNV-CP/*EcSnx27* complex (Fig. 3C; Table S4). The diffused and aggregated fluorescence distribution of *EcSnx27* (GFP) almost overlapped with that of RGNNV-CP (RFP), indicating the colocalization of *EcSnx27* and RGNNV-CP (Fig. 3D). Together, our data suggested that *EcSnx27* could interact with RGNNV-CP.

To explore the key domain responsible for the interaction between EcSNX27 and RGNNV-CP, we then developed different truncations (Fig. 3E). The four functional domains present in *EcSnx27* were identified as the SNX-PDZ, SNX-PX, SNX-FERM, and SNX-FERM-like domains. Truncated versions of the protein lacking one domain at a time were created (Fig. 3E). We then expressed these truncated versions of *EcSnx27* in GK cells and performed interaction studies with RGNNV-CP after virus infection. We employed a Co-IP assay to assess the interaction between truncated *EcSnx27* and RGNNV-CP. The deletion of either SNX-FERM or SNX-FERM-like domains resulted in a loss of interaction (Fig. 3F) and co-localization (Fig. S1) with RGNNV-CP compared to full-length *EcSnx27* and other mutations. The *in silico* analysis also revealed that 8 of the 18 hydrogen bonds between RGNNV-CP and *EcSnx27* were associated with the residues within the FERM and FERM-like domains of the *EcSnx27* protein (Table S4). Accordingly, the SNX-FERM and SNX-FERM-like domains were considered key domains for this interaction. Additionally, we found that the SNX-PX domain was responsible for the subcellular localization of *EcSnx27* (Fig. 3G).

### RGNNV infection increases exosome release from GK cells

**Fig 4:**
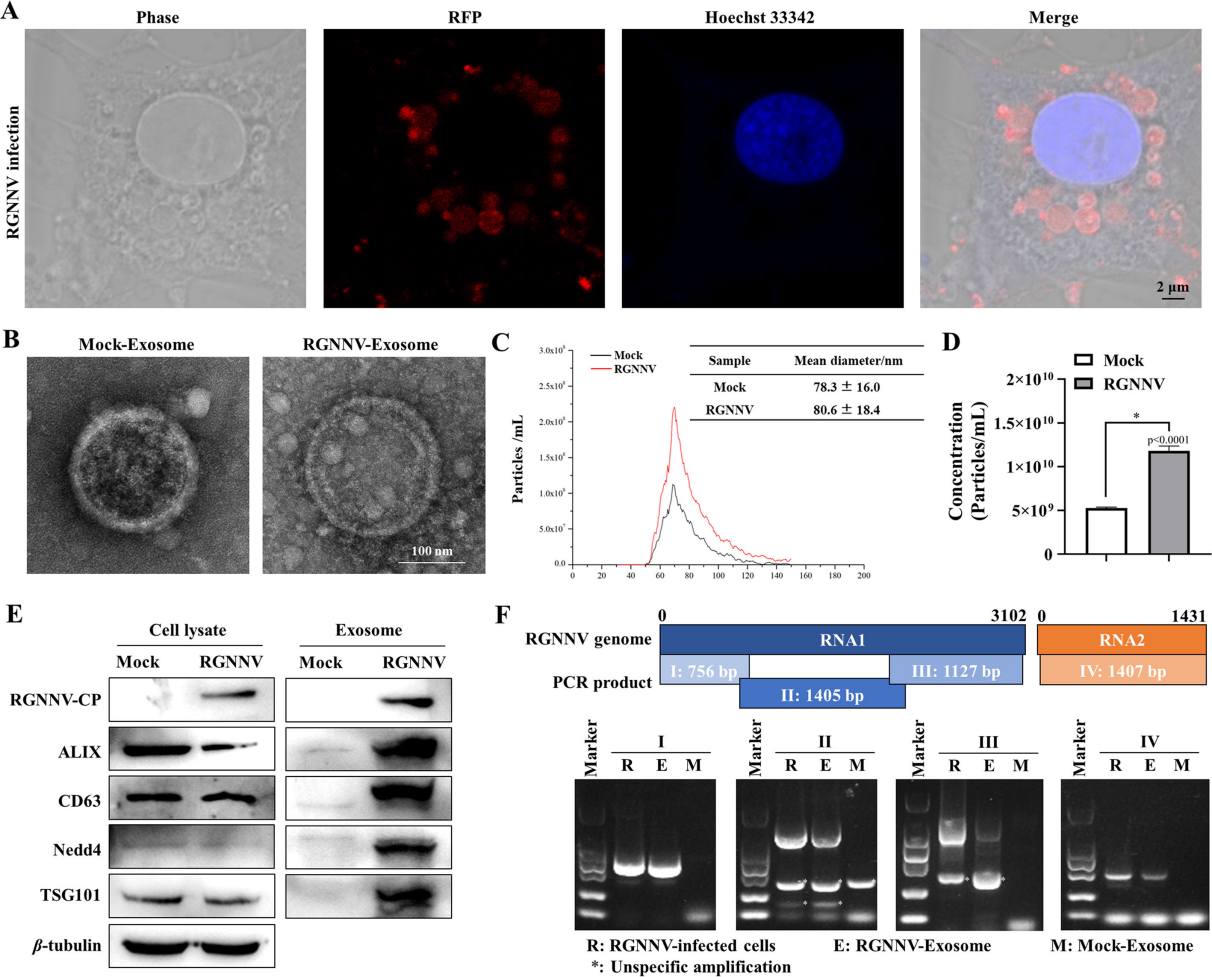
RGNNV infection promotes exosome production. (**A**) The IFA suggested that RGNNV virions were contained within the intracellular vesicles. GK cells seeded in a glass-bottom dish were infected with RGNNV at an moi of 2.0. After 48 h p.i., cells were fix, and IFA was performed using anti-RGNNV-CP antibody. The nuclei were stained with Hoechst33342. Samples were imaged using a confocal microscope (Olympus). (**B**) Exosomes were observed using TEM after mock or RGNNV infection. GK cells were seeded in 10-cm dishes followed by mock or RGNNV infection. Cells were washed with PBS buffer after 6 h p.i., and the cell cultures were replaced with fresh L-15 medium supplemented with 10% exosome-depleted FBS. The cell cultures were collected after 48 h p.i. The extracted exosomes were used for TEM and NanoSight analysis. (**C, D**) The exosomes released from GK cells after mock or RGNNV infection were analyzed by NTA assay. RGNNV infection increased the diameter and concentration of the exosomes extracted. (**E**) Exosome-associated marker proteins were examined from the cell lysate and exosomes in the mock- or RGNNV-infected groups. Higher levels of the exosome-associated proteins were observed in the RGNNV group. (**F**) Four overlapping fragments (I, II, III in RNA1, and IV in RNA2) were designed based on the genome sequence of RGNNV, and RGNNV genomic RNA in exosomes isolated from RGNNV-infected cells was detected with RT-PCR. The data are presented as mean ± SD, *n* = 3. The significance of the variability between the different treatment groups was calculated with the two-tailed, unpaired Student *t*-test, *P < 0.05.

It has been revealed that RGNNV coat proteins were present within intracellular vesicles as shown in the IFA image (Fig. 4A); consequently, it is imperative to determine whether RGNNV infection involves the exosome-related pathway. As a consequence, we isolated the exosomes following the manufacturer’s instructions, and exosomes from mock- or RGNNV-infected cells were subjected to transmission electron microscopy (TEM; Fig. 4B) and nanoparticle tracking analysis (NTA; Fig. 4C). Consistent with TEM data, exosomes isolated from mock-infected cells had a mean particle size of 78.3 ± 16.0 nm in NTA, whereas exosomes isolated from RGNNV-infected cells had mean particle sizes of 80.6 ± 18.4 nm, suggesting that RGNNV infection does not significantly change the exosome size in GK cells. Interestingly, RGNNV infection significantly increased the number of the exosomes released from GK cells (Fig. 4D). Moreover, the marker proteins in the cell lysate and exosomes extracted from mock- or RGNNV-infected GK cells were detected using western blotting. The RGNNV infection significantly increased the formation of exosomes (Fig. 4C and D), and subsequently, the expression of exosome-related proteins, including ALIX, CD63, Nedd4, and TSG101, was increased as well (Fig. 4E). To further explore whether RGNNV-associated exosomes carry complete viral genomic RNA, we employed the RT-PCR to amplify four fragments (I, II, III, and IV) that encompass the entire RGNNV genome. Our results demonstrated that all overlapping fragments were detectable in the exosomes extracted from RGNNV-infected cells (Fig. 4F). Collectively, these findings suggest that high-purity RGNNV-associated exosomes can be isolated through immunoblotting and contain full-length viral genomic RNAs.

### *EcSnx27* overexpression promoted virus sorting into the exosomes

**Fig 5:**
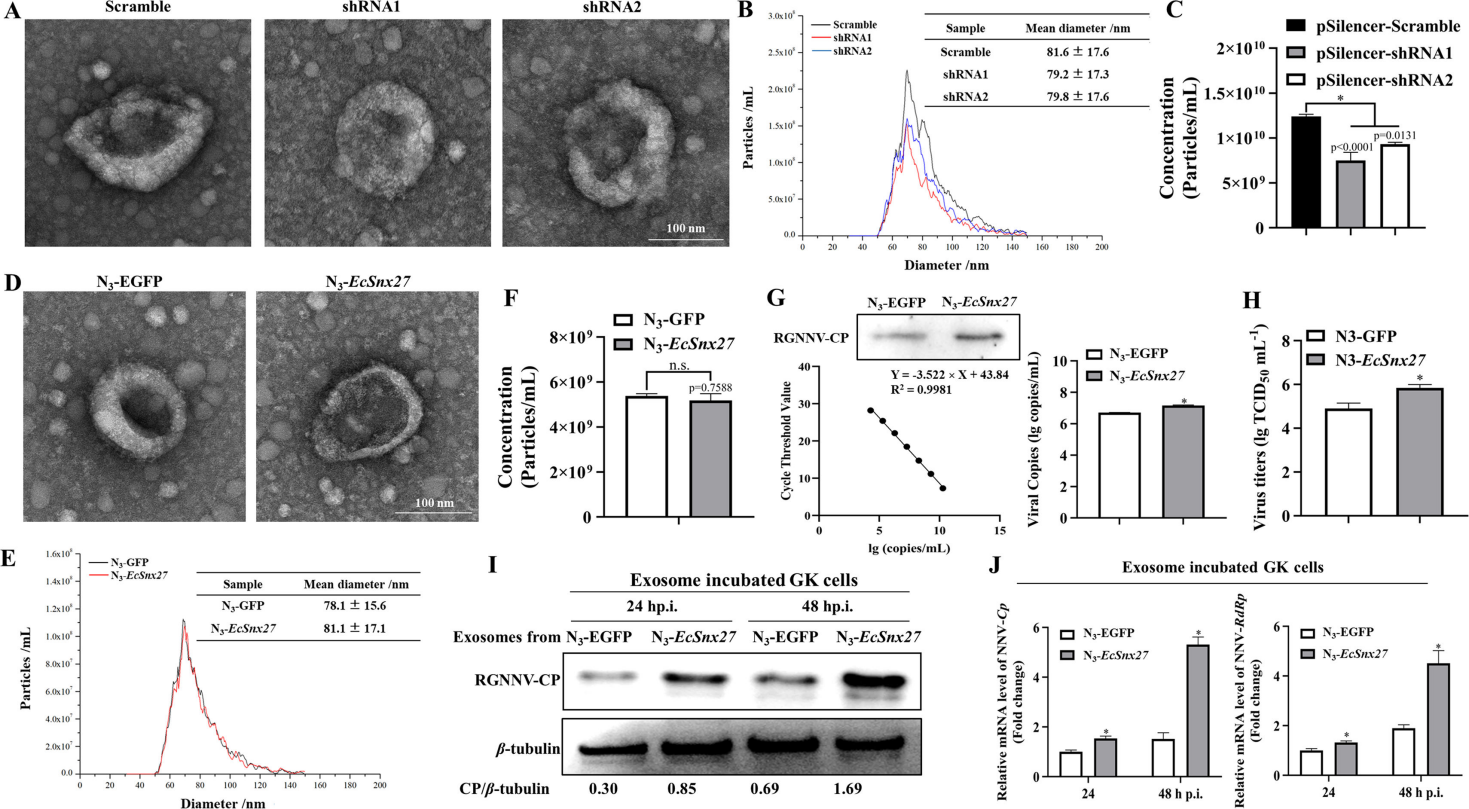
(**A**) Exosomes in the supernatants of indicated plasmid-transfected GK cells were purified by the Total Exosome Isolation Reagent and analyzed by TEM. (**B**) NTA shows the absolute number of exosomes per milliliter of cell culture supernatants from indicated groups. (**C**) The concentration of the exosomes reduced significantly after *EcSnx27* knockdown. (**D**) Exosomes extracted from the supernatants of N_3_-GFP- or N_3_-*EcSnx27*-transfected cells were analyzed using TEM. (**E, F**) NTA suggests that overexpression of *EcSnx27* did not affect exosome concentration. (**G, H**) RGNNV coat protein and viral copies from the exosomes isolated from the supernatants of N_3_-GFP- or N_3_-*EcSnx27*-transfected cells were analyzed through western blotting and absolute qPCR. GK cells infected with RGNNV at an moi of 1.0 (30 min at 4℃) were washed with PBS three times before incubated with FBS-free L-15 at 28℃ for virus entry. Then, the cells were transfected with N_3_-GFP or N_3_-*EcSnx27* plasmids at 4 h p.i., respectively. At 6 h post transfection, the culture medium was changed with fresh L-15 (10% exosome-depleted FBS contained). The exosomes were isolated at 24 h p.i., the containing viral coat protein and RNAs were analyzed (**G**), and the virus titer of the exosomes were also detected through TCID_50_ (**H**). (**I, J**) The extracted exosomes were used to incubate GK cells. Cells were collected at indicated time points for western blotting (**I**) and qPCR analysis (**J**). The data are presented as mean ± SD, *n* = 3. The significance of the variability between the different treatment groups was calculated with the two-tailed, unpaired Student *t*-test, *P < 0.05.

The knockdown of *EcSnx27* led to a decreased number of exosomes released from GK cells, though the diameter of the exosomes remained unchanged (Fig. 5A through C), while the overexpression of *EcSnx27* has no impact on the diameter and concentration of exosomes (Fig. 5D through F). The contained virions within the exosomes extracted from the control or *EcSnx27*-overexpressing culture media were detected through western blot and absolute qPCR. As revealed in Fig. 5G, overexpression of *EcSnx27* increased the level of viral CP and viral RNAs in the exosomes (Fig. 5G). The data suggested that *EcSnx27* improved the secretion of important viral components. Then, the exosomes extracted from N_3_-GFP- or N_3_-*EcSnx27*-transfecting groups were used to infect GK cells. More virus products (Fig. 5E and F) and higher virus titers (Fig. 5G) were detected from the *EcSnx27* overexpression group than those from the control group. Together, these results revealed that *EcSnx27* overexpression did not affect the exosome formation but increased the virion containing within the exosomes.

### RGNNV infection involved with ALIX-mediated exosome secretion

**Fig 6:**
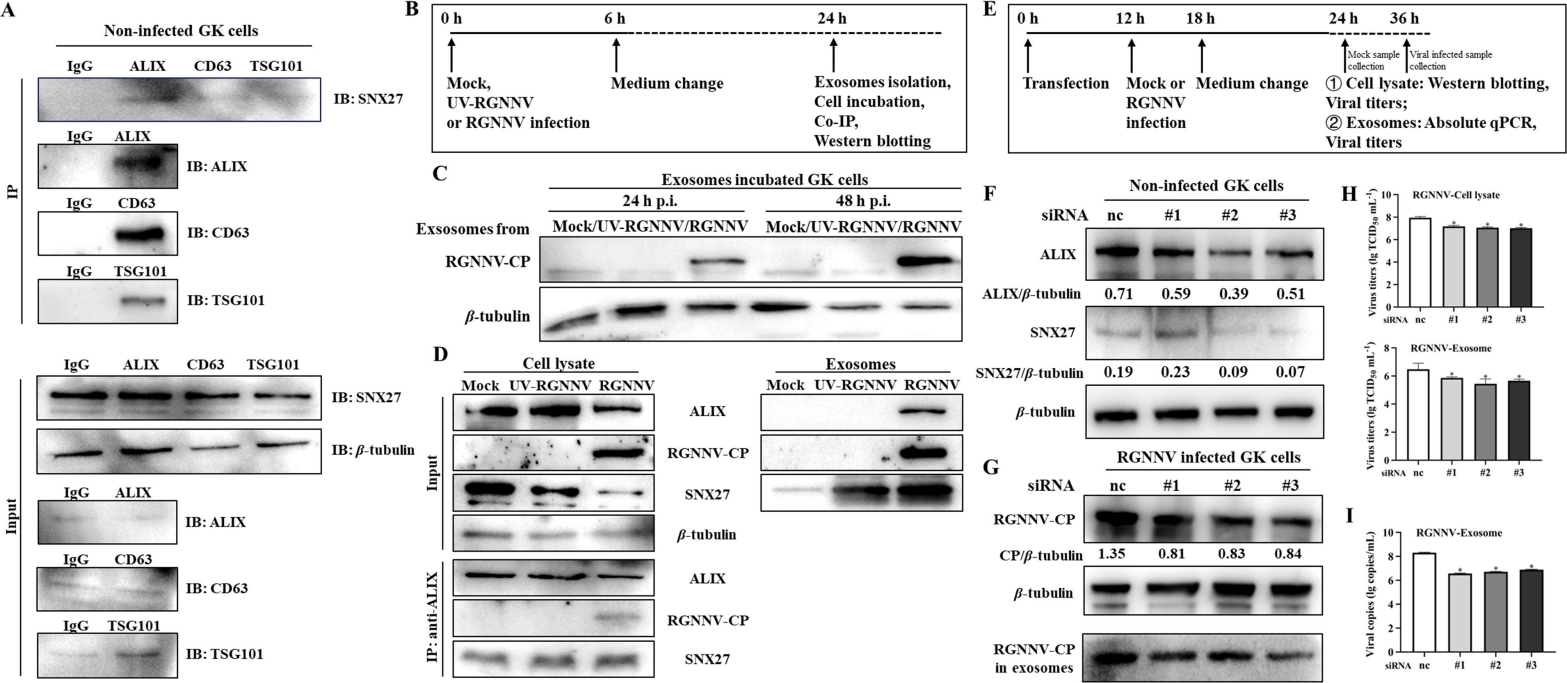
ALIX plays a role in forming the RGNNV-CP/*EcSnx27*/ALIX complex. (**A**) To evaluate the potential ESCRT-related protein that could interact with *Snx27*, the anti-ALIX, anti-CD63, and anti-TSG101 antibodies were used for the Co-IP assay. IgG was used as the control. (**B**) Experimental scheme. GK cells were infected with mock, UV-RGNNV or RGNNV, respectively. The cell cultures were replaced with fresh L-15 medium supplemented with 10% exosome-depleted FBS. The exosomes were isolated at 24 h p.i. and stored in −80℃ for further use. (**C**) Exosomes isolated from the mock, UV-RGNNV, or RGNNV group were used to incubate GK cells. After 24 and 48 h post incubation, GK cells were collected, and the cell lysate was used for western blotting using anti-RGNNV-CP antibody and *β*-tubulin. (**D**) After mock-, UV-RGNNV, or RGNNV infection, GK cells were collected for IP analysis using anti-ALIX antibody. RGNNV-CP could be immunoprecipitated with ALIX. The exosomes were also extracted from the culture medium of each group, and the protein level of ALIX, RGNNV-CP, and SNX27 were detected. Compared with the protein levels in cell lysate, RGNNV infection facilitated the secretion of SNX27 in the exosomes. Accordingly, RGNNV-CP, *EcSnx27,* and ALIX may form a complex in fish cells. (**E**) Experimental scheme. GK cells were transfected with small interfering RNA. After RGNNV infection for 6 h, the cell cultures were replaced with fresh L-15 medium supplemented with 10% exosome-depleted FBS. The exosomes were isolated, and the cells were sampled at 24 h post transfection or post infection and stored in −80℃ for further use. (**F**) Knockdown of ALIX reduced the protein level of *Snx27* intracellularly. (**G**) Knockdown of ALIX reduced the protein level of RGNNV-CP in cells and exosomes. The total protein levels in the exosomes were quantified using BCA protein assay kit before western blotting. (**H**) Knockdown of ALIX reduced the viral titers in both cells and exosomes. (**I**) Knockdown of ALIX reduced the viral copies in the exosomes. The data are presented as mean ± SD, *n* = 3. The significance of the variability between the different treatment groups was calculated with the two-tailed, unpaired Student *t*-test, *P < 0.05.

To ascertain the exosome-related protein that interacts with *EcSnx27*, GK cells were seeded in 10-cm dishes and infected with RGNNV at an moi of 1.0. After 24 h p.i., IP assay was performed using anti-ALIX, anti-CD63, and anti-TSG101 antibodies. The IgG was used as a control. The ALIX interacted with *EcSnx27* (Fig. 6A); accordingly, we speculated that ALIX interacted with *EcSnx27* and sorted the complex of ALIX*–EcSnx27*–RGNNV-CP into the exosomal pathway, benefiting virus release. To support our hypothesis, mock, UV-RGNNV (explored in ultraviolet irradiation at 150 W for 30 min), or RGNNV was used to infect GK cells, and then the exosomes were extracted from the cell culture for further analysis (Fig. 6B). The extracted exosomes were used for the incubation of GK cells. The western blot was employed to evaluate viable virions contained within the exosomes. No viable viruses were detected within the mock or UV-RGNNV-derived exosomes (Fig. 6C), consistent with the results of non-detected ALIX (Fig. 6D, input pattern), we assumed that only viable virions could trigger the ALIX-associated exosomal pathway. Co-IP assay also revealed that RGNNV-CP could interact with ALIX (Fig. 6D, IP pattern). No interaction between UV-RGNNV-CP and ALIX suggested that ALIX might not interact with the viral CPs from the initial infection (Fig. 6D). Our results suggested that the ALIX might sort the progeny virus or newly assembled viral CP into exosomes. The ALIX-associated endosomal sorting complexes required for transport machinery (ESCRT)-dependent pathway may be hijacked by the RGNNV infection.

Small interfering RNAs (siRNAs) for ALIX knockdown (Table S2) were synthesized from the Genecreate Biological Engineering Co., Ltd. As shown by western blotting, the knockdown of ALIX reduced the expression of intracellular or exosomal SNX27 (Fig. 6F). The interfering of ALIX also decreased RGNNV replication (Fig. 6G) and virus product (Fig. 6G through I). Hence, ALIX is critical for RGNNV product.

**Fig 7:**
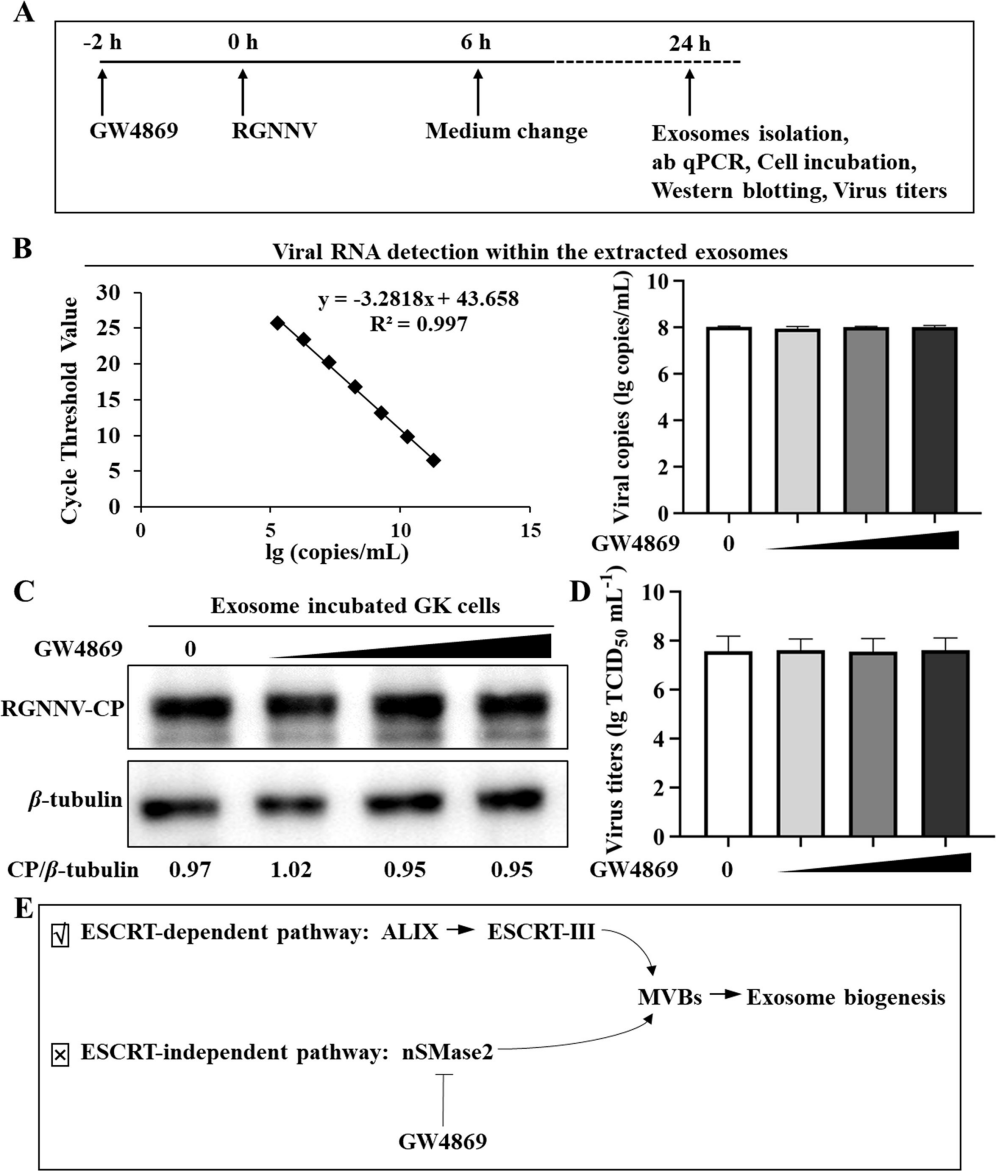
The exosome inhibitor GW4869 had no impact on RGNNV production. (A) Experimental scheme for GW4869 treatments. GK cells were treated with doses of GW4869 at a final concentration of 0, 2, 5, or 10 µM before infected with RGNNV (at an moi of 1.0). After infection for 6 h, the cultures were washed and treated with the same doses of GW4869 in fresh medium for another 18 h. Then, the exosomes were isolated for further analysis. (B) Viral RNA contained within the extracted exosomes were evaluated using absolute qPCR. (C) GK cells were incubated with the extracted exosomes for 48 h. After cell collection, the samples were analyzed by western blotting using anti-RGNNV-CP and anti-*β*-tubulin antibodies. (D) GK cells were treated with GW4869 and RGNNV as described previously. Subsequently, exosomes were isolated from the cell culture, and the viral titers in the extracted exosomes were assessed using the TCID_50_ assay. The data are presented as mean ± SD, *n* = 3. The significance of the variability between the different treatment groups was calculated with the two-tailed, unpaired Student *t*-test, *P < 0.05. (E) RGNNV infection induced ESCRT-dependent pathway.

Exosomes are released from intracellular structures known as multi-vesicular bodies (MVBs). The formation of MVBs involves two distinct mechanisms: the ESCRT-dependent pathway and the ESCRT-independent pathway (40). Previous results revealed that the ALIX, which also functioned as the intermediate of the ESCRT was involved in RGNNV infection. Thus, RGNNV induced exosome release through the ESCRT-dependent pathway. To further understand whether the ESCRT-independent pathway affects RGNNV production, the GW4869, a potent neutral sphingomyelinase (nSMase) inhibitor that prevents the formation of ILVs and blocking exosome production through the ESCRT-independent pathway, was employed. GK cells were treated with GW4869 at a final concentration of 0, 2, 5, or 10 µM before infected with RGNNV (at an moi of 1.0). After 6 h of infection, the cell cultures were replaced with fresh medium supplemented with the same doses of GW4869. Cells were incubated for another 18 h. Then, the exosomes were isolated for absolute qPCR analysis and cell incubation. As a result, GW4869 had no impact on the RGNNV production in the extracted exosomes, as evidenced by the absolute qPCR analysis (Fig. 7B), western blotting (Fig. 7C), and viral titers (Fig. 7D). Accordingly, GW4869 acts as an exosome inhibitor in the endocytic pathway but has no impact on RGNNV replication. Together, RGNNV may hijack *EcSnx27* and modulate the ALIX-associated ESCRT pathway (Fig. 7E).

## DISCUSSION

This study investigated the function of *EcSnx27* in immune modulation and RGNNV infection. *EcSnx27* negatively regulated the IFN pathway by inhibiting *Irf3* and subsequently promoting RGNNV replication. Moreover, the interactions between *EcSnx27*, RGNNV-CP, and ALIX suggest that *EcSnx27* affects the transport of RGNNV virions in fish cells. Furthermore, the increased number of exosomes released from RGNNV-infected cells revealed that RGNNV infection affects the exosomal pathway. The *EcSnx27* can interact with ALIX and increase the infection of the exosomes extracted. This indicates that *EcSnx27* aids in the viral release process through exosomal machinery.

Studies have suggested that *Snx25* and *Snx27* inhibit the NF-κB signal (27, 41). Our study presented similar results; *EcSnx27* downregulated both the protein and transcription levels of NF-κB. According to previous studies, NF-κB regulation manner in fish cells might differ from that in mammalian cells (27, 41). In addition, SNX proteins are also involved in the IFN pathway (26, 30). The *Snx8* from grass carp could negatively regulate the IFN response induced by the RLR-mediated innate immune response by directly binding to the stimulator of interferon gene (STING) (42), whereas the *Snx8* from mammals functions as a positive regulator of the innate immune response through its interaction with virus-induced signaling adaptor (VISA) (43). Limited evidence suggests that *Snx27* can affect the IFN pathway. However, our findings confirmed that fish *Snx27* inhibits IFN signaling by reducing the transcription and translation levels of IFN-related molecules in lower vertebrate cell lines. Furthermore, the decreased ISRE and NF-κB promoter activity indicated that *EcSnx27* negatively regulates fish innate immunity. Consequently, *EcSnx27* may promote RGNNV replication through its negative regulation of the NF-κB and IFN pathways.

The IFN system, with a potent antiviral function in the host immune response, is usually disrupted by the virus as an immune evasion strategy (44). Viruses reportedly antagonize type I IFN production to facilitate viral replication in multiple ways, including autophagy and ubiquitination (45, 46). As for the RGNNV, virus infection triggers RLR-mediated IFN activation in different kinds of fish cells (47, 48). We have shown that *EcSnx27* suppresses IFN-related cytokines and potentially benefits RGNNV infection. Our study showed that the *Irf3* may be a target of *EcSnx27* in regulating the IFN system. However, no signs of direct interaction between *EcSnx27* and RLRs were indicated by either IP-MS or Co-IP analyses (data not shown). Further studies are needed to uncover the underlying mechanisms of *EcSnx27* in IFN modulation.

The retromer complex is a multi-protein assembly that recycles proteins from endosomes to the trans-Golgi network or plasma membrane, thereby regulating their levels and spatial distribution within cells (49). As a retromer protein, *Snx27* regulates protein trafficking from the early endosome to the plasma membrane (50). It has been demonstrated that the interaction of *Snx27* and the L2 minor capsid protein of human papillomavirus (HPV) is involved with viral genome trafficking and promoting viral infection and replication (51, 52). The results of the interaction between *EcSnx27* and RGNNV-CP, as evidenced by the Co-IP assay and molecular docking results, as well as the colocalization of the protein with the viral coat protein, all revealed that *EcSnx27* might impact viral protein transport in cells. Three functional domains have been characterized in *EcSnx27*, including SNX-PDZ, SNX-PX, and two SNX-FERM domains. The SNX-PDZ domain is an important protein–protein interaction module involved in various biological processes, including ion channel formation, protein transport, signal transduction, and cell adhesion (53). Human immunodeficiency virus I (HIV-1)-encoded nucleocapsid mimics the PDZ domain to bind to ALIX and promote virus budding (54). The role of the SNX-PDZ domain during RGNNV infection was not determined in this study. We hypothesized that the interaction between *EcSnx27* and ALIX may be involved in the function of the SNX-PDZ domain. Evidence suggests that the SNX-PX domain is sufficient for binding the SNX protein to the endosomal membrane (55, 56). A comparable result has been observed that the deletion of the SNX-PX domain in the *EcSnx27* weakened the protein’s location, implying that SNX-PX is responsible for the subcellular localization of *EcSnx27*. Co-immunoprecipitations have confirmed that *EcSnx27* interacts with RGNNV-CP through the SNX-FERM and SNX-FERM-like domains. The simultaneous presence of SNX-FERM and SNX-FERM-like domains was essential for this interaction. Furthermore, the knockout of either functional domain would impair the full functionality of *EcSnx27*, as indicated by the decreased viral production. In summary, all the domains were of great importance for *EcSnx27*. In this study, we characterized *EcSnx27*, which acted as a linker by grabbing RGNNV-CP with the SNX-FERM and SNX-FERM-like domains, and perhaps binding to the ESCRT complex protein ALIX through the SNX-PDZ domain, subsequently modulating viral transport.

Exosomes released from almost all virus-infected cells are important during viral infection (22, 57). Numerous viruses interact with ESCRT-related proteins and utilize the exosome/extracellular vesicle generation pathway to facilitate viral replication (58). The ESCRT machinery regulates EBV maturation during viral replication (59). The exosomal machinery plays a role in HIV-1 viral RNA and protein trafficking (60). The non-structural protein 3A of enterovirus 71 binds to vacuolar protein sorting 25 and promotes viral replication by boosting exosome biogenesis (22). In RGNNV infection, most virions and viral RNA are located within cytoplasmic vacuoles (61, 62). However, the formation, progression, and mechanism of RGNNV-induced vacuolization remains unclear. Our data indicated that RGNNV coat protein and full-length genomic RNAs could be detected in the exosomes extracted from the supernatants of virus-infected cells. Combined with the infectivity of the virus-associated exosomes, we proposed for the first time that the exosomal pathway may be associated with the release of RGNNV. RGNNV might modulate the exosomal machinery to facilitate viral products by interacting with *EcSnx27*. As a result, *EcSnx27* is the key factor hijacked by RGNNV. The interaction of ALIX with *EcSnx27* and RGNNV-CP proposes that they might form a complex and poses an important clue that the ALIX-mediated exosomal machinery might be of great significance during RGNNV infection. The knockdown of ALIX resulted in a reduction of both *Snx27* and viral products in cells and exosomes, suggesting that the ALIX-associated ESCRT pathway is essential for RGNNV infection. On the other hand, the nSMase inhibitor GW4869 had no impact on RGNNV replication, indicating that RGNNV infection may not involve an nSMase-mediated manner. Thus, RGNNV might utilize the ALIX-mediated exosomal pathway during viral infection.

In conclusion, our data suggest that the *Snx27* gene encoded by the orange-spotted grouper could inhibit IFN response and potentiate the transport and release of RGNNV through the ALIX-mediated exosomal pathway, thereby facilitating viral products. We believe that our observations provide a novel mechanism by which *Snx27*-mediated cell signaling and protein trafficking affect viral replication and are potential targets for antiviral applications.

## Data Availability

The data supporting this study are openly available as follows: the GenBank accession numbers of RGNNV genomic sequences are MW265973.1 (RNA1) and MW265974.1 (RNA2); the obtained sequences of *EcSnx27* and EcALIX have been deposited in GenBank under accession numbers PQ275721 and PQ275722, respectively.

## SUPPLEMENTAL MATERIAL

Fig. S1 jvi.00974-24-s0001.docx The FERM and FERM-like domains were of great significance for the co-localization between RGNNV-CP and *EcSnx27*.

Table S1 jvi.00974-24-s0002.docx Primers used in this study.

Table S2 jvi.00974-24-s0003.docx Sequences of the shRNA and siRNA used in this study.

Table S3 jvi.00974-24-s0004.docx Similarities of amino acid sequences of *Snx27* between the red-spotted grouper and other vertebrates.

Table S4 jvi.00974-24-s0005.docx The formation of the hydrogen bond residues and distances between *EcSnx27* and RGNNV-CP.

ASM does not own the copyrights to Supplemental Material that may be linked to, or accessed through, an article. The authors have granted ASM a non-exclusive, world-wide license to publish the Supplemental Material files. Please contact the corresponding author directly for reuse.
