# Supplementary material for: Fish Snx27 promotes viral products by modulating the innate immune response and exosomal machinery
Source: J Virol. 2024 Nov 4;98(12):e00974-24. doi: 10.1128/jvi.00974-24 (PMC11650975; doi:10.1128/jvi.00974-24)
Supplement: Fig. S1 — The FERM and FERM-like domains were of great significance for the co-localization between RGNNV-CP and EcSnx27. [file jvi.00974-24-s0001.docx]

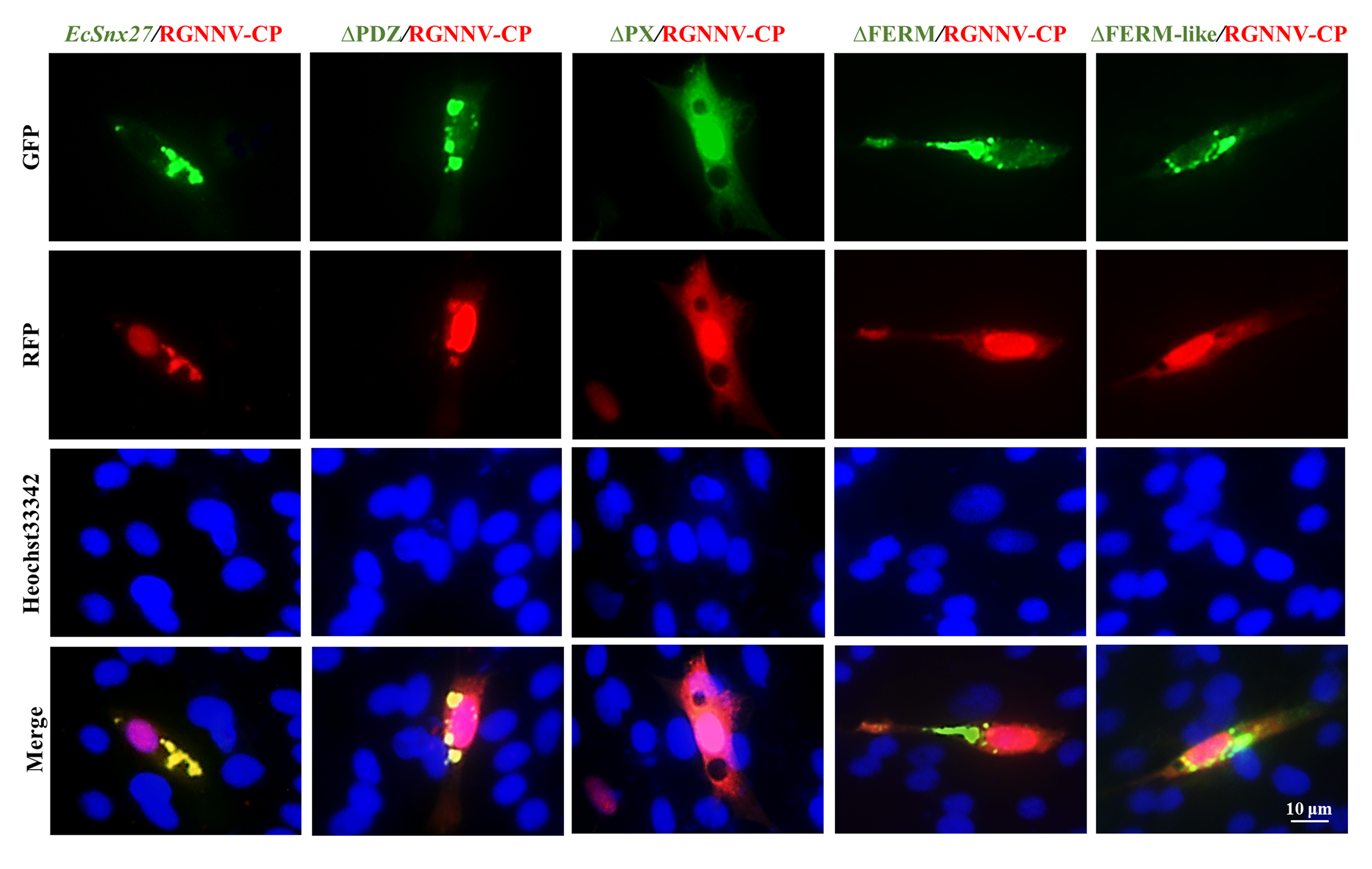


**Fig. S1** The FERM and FERM-like domains were of great significance for the co-localization between RGNNV-CP and *EcSnx27*. 3HA-Cp plasmid was co-transfected with N3-*EcSnx27*, N3-∆PDZ, N3-∆PX, N3-∆FERM or N3-∆FERM-like into GK cells at a 1:1 ratio, respectively. Cells were fixed 48 h post transfection, and the IFA was carried out using anti-HA antibody (Cell signaling, Cat No.: 2367) and Goat Anti-Mouse IgG H&L (Abcam, Cat No.: ab150116) antibodies following by nuclei staining using Heochst33342 and imaged by EVOS FL Auto (Life technologies). Green signals represent GFP-EcSnx27 and its mutations, red signals represent RGNNV-CP, blue signals represent nuclei, and yellow signals in merged images represent the co-localization. Scale bars are shown as 10 μm.
