# Supplementary material for: Fish Snx27 promotes viral products by modulating the innate immune response and exosomal machinery
Source: J Virol. 2024 Nov 4;98(12):e00974-24. doi: 10.1128/jvi.00974-24 (PMC11650975; doi:10.1128/jvi.00974-24)
Supplement: Table S1 — Primers used in this study. [file jvi.00974-24-s0002.docx]

| **Usage** | **Name** | **Sequences (5’-3’)** |
| --- | --- | --- |
| PCR and sequencing | *EcSnx27*-seq-PF | ATGGCGAATGTAGGAGACG |
|  | *EcSnx27* -seq-PR | TTAGGAAGTTGCAATCTCTCC |
|  | RGNNV-RNA1-I-PF | TAACATCACCTTCTTGCTCTG |
|  | RGNNV-RNA1-I-PR | CAAGATCTGCATCAGATTCG |
|  | RGNNV-RNA1-II-PF | ACCGCATCTGGAACTACAACC |
|  | RGNNV-RNA1-II-PR | CCAGCAAGGTATGAGACGAAA |
|  | RGNNV-RNA1-III-PF | CACCTGCTGTTACACGATT |
|  | RGNNV-RNA1-III-PR | GCATAAAGCTGATTAAGGGA |
|  | RGNNV-RNA2-IV-PF | CAATGGTACGCAAAGGTGAG |
|  | RGNNV-RNA2-IV-PR | CGAGTTGAGAAGCGATCAGC |
| Vector construction | *EcSnx27*-GFP-PF | CAAGTCCGGACTCAGATCTCGAGCTATGGCGAATGTAGGAGACG |
|  | *EcSnx27*-GFP-PR | GGATCCCGGGCCCGCGGTACCGGAAGTTGCAATCTCTCCC |
|  | shRNA-PF | GAGCTGGTTTAGTGAACCGTGGATCCTGCTGTTGACAGTGAGCG |
|  | shRNA-PR | CCAATGAAAATAAAAGATCCTTTATTAAGCTTTCCGAGGCAGTAGGCA |
|  | RGNNV-CP-PF | CTTAAGCTTGGTACCGAGCTCGGATCCATGGTACGCAAAGGTGAGAAGAAAT |
|  | RGNNV-CP-PR | TCGTATGGGTAGGGCCCTCTAGACTCGAGGTTTTCCGAGTCAACCCTGGTG |
|  | ΔPDZ-PR-upper | CAGCCTCCTGAGCTGGAACAGAACCTGACGTTACCATAACGC |
|  | ΔPDZ-PF-lower | GCGTTATGGTAACGTCAGGTTCTGTTCCAGCTCAGGAGGC |
|  | ΔPX-PR-upper | GTCACTCCATTGTAGTTCTCATCGTCACTGTAGTCATAGTTGGGTTGG |
|  | ΔPX-PF- lower | CAACCCAACTATGACTACAGTGACGATGAGAACTACAATGGAGTGACAG |
|  | ΔFERM-PR-upper | GTCTCTCAGCAACTCCTCCTCCACTCCATTGTAGTTCTCATCTG |
|  | ΔFERM-PF-lower | GATGAGAACTACAATGGAGTGGAGGAGGAGTTGCTGAGAGAC |
|  | ΔFERM-like-PR-upper | GTCAGATGTTTCTTCCTCCACGTAGCCCTCGCATGTTCGC |
|  | ΔFERM-like-PF-lower | GTTGCGAACATGCGAGGGCTACGTGGAGGAAGAAACATCTGAC |
| RT-PCR analysis | *EcSnx27*-RT-PF | GCTCCGCAAATGGTTGTT |
|  | *EcSnx27*-RT-PR | GCGTGCAGCTTGAAGTGA |
|  | *Ef1α*-RT-PF | AAAGAGCGATGTCAATGGTG |
|  | *Ef1α*-RT-PR | GATCTACAAGTGCGGAGGAAT |
|  | *EcIrf3*-RT-PF | ATGGTTTAGATGTGGGGGTGTCGGG |
|  | *EcIrf3*-RT-PR | GAGGCAGAAGAACAGGGAGCACGGA |
|  | *EcIrf7*-RT-PF | CAACACCGGATACAACCAAG |
|  | *EcIrf7*-RT-PR | GTTCTCAACTGCTACATAGGGC |
|  | *EcTbk1*-RT-PF | CCTGCTGACCGACAACTGGA |
|  | *EcTbk1*-RT-PR | GAGGCGATATTTCATGGCACA |
|  | *EcIsg15*-RT-PF | CCTATGACATCAAAGCTGACGAGAC |
|  | *EcIsg15*-RT-PR | GTGCTGTTGGCAGTGACGTTGTAGT |
|  | *EcMx1*-RT-PF | CGAAAGTACCGTGGACGAGAA |
|  | *EcMx1*-RT-PR | TGTTTGATCTGCTCCTTGACCAT |
|  | *NF-κB*-RT-PF | GGCTAAAGAAATGGACCTCA |
|  | *NF-κB*-RT-PR | CATAGAACCGAACCTGGATA |
|  | RGNNV-*Cp*-PF | CAACTGACAACGATCACACCTTC |
|  | RGNNV-*Cp*-PR | CAATCGAACACTCCAGCGACA |
|  | RGNNV-*Rdrp*-PF | GTGTCCGGAGAGGTTAAGGATG |
|  | RGNNV-*Rdrp*-PR | CTTGAATTGATCAACGGTGAACA |

Supplementary Table1. Primers used in this study
