# Supplementary material for: Fish Snx27 promotes viral products by modulating the innate immune response and exosomal machinery
Source: J Virol. 2024 Nov 4;98(12):e00974-24. doi: 10.1128/jvi.00974-24 (PMC11650975; doi:10.1128/jvi.00974-24)
Supplement: Table S2 — Sequences of the shRNA and siRNA used in this study. [file jvi.00974-24-s0003.docx]

| **Usage** | **Name** | **Sequences (5’-3’)** |
| --- | --- | --- |
| shRNA sequence | *EcSnx27*-shRNA1 | TGCTGTTGACAGTGAGCGAGGATAGCTCTGCCAGACAAGATAGTGAAGCCACAGATGTATCTTGTCTGGCAGAGCTATCCGTGCCTACTGCCTCGGA |
|  | *EcSnx27*-shRNA2 | TGCTGTTGACAGTGAGCGAGCAAATGGTTGTTCAGCTTCCTAGTGAAGCCACAGATGTAGGAAGCTGAACAACCATTTGCGTGCCTACTGCCTCGGA |
|  | Scramble sequence | TGCTGTTGACAGTGAGCGAAGCATCTAAGGCGACCTCGTTAGTGAAGCCACAGATGTAGGTCAGTAGGTCAACCTCCAGTGCCTACTGCCTCGGA |
| ALIX siRNA | Negative control | Sense (5'-3'): ACGUGACACGUUCGGAGAATT  Antisense (5'-3'): UUCUCCGAACGUGUCACGUTT |
|  | siRNA#1 | Sense (5'-3'): AGACGUGCGUGUUGUUCAATT  Antisense (5'-3'): UUGAACAACACGCACGUCUTT |
|  | siRNA#2 | Sense (5'-3'): AGCUGAGAACCAAGUUCAATT  Antisense (5'-3'): UUGAACUUGGUUCUCAGCUTT |
|  | siRNA#3 | Sense (5'-3'): GCGAAGGCACCAAGUUCUATT  Antisense (5'-3'): UAGAACUUGGUGCCUUCGCTT |

Supplementary Table2. Sequences of the shRNA and siRNA used in this study
