# Supplementary material for: Fish Snx27 promotes viral products by modulating the innate immune response and exosomal machinery
Source: J Virol. 2024 Nov 4;98(12):e00974-24. doi: 10.1128/jvi.00974-24 (PMC11650975; doi:10.1128/jvi.00974-24)
Supplement: Table S3 — Similarities of amino acid sequences of Snx27 between the red-spotted grouper and other vertebrates. [file jvi.00974-24-s0004.docx]

**Supplementary Table 3** Similarities (%) of amino acids sequences (full-length and/or functional domains) of *Snx27* between the Red-spotted grouper and other vertebrates.

|  | Access number | Full-length | PDZ | PX | FERM | FERM-like |
| --- | --- | --- | --- | --- | --- | --- |
| *Epinephelus lanceolatus lanceolatus* | XP_033492583.1 | 99.65 | 100 | 100 | 100 | 100 |
| *Perca flavescens* | XP_028453502.1 | 98.41 | 98.92 | 100 | 98.92 | 98.04 |
| *Dicentrarchus labrax* | XP_051245963.1 | 96.99 | 98.92 | 100 | 98.92 | 97.06 |
| *Scomber japonicus* | XP_053190351.1 | 95.40 | 97.85 | 100 | 98.92 | 96.08 |
| *Scomber scombrus* | XP_062291758.1 | 95.22 | 97.85 | 99.06 | 98.92 | 97.06 |
| *Pleuronectes platessa* | XP_053302546.1 | 93.84 | 98.92 | 96.23 | 98.92 | 99.02 |
| *Danio rerio* | NP_001038565.1 | 86.27 | 94.62 | 94.34 | 93.41 | 90.20 |
| *Rana temporaria* | XP_040188386.1 | 83.76 | 95.70 | 89.62 | 85.71 | 90.20 |
| *Mus musculus* | NP_001075953.1 | 85.19 | 93.55 | 90.57 | 82.42 | 92.16 |
| *Homo sapiens* | NP_001317652.1 | 84.36 | 92.47 | 89.62 | 80.22 | 92.16 |
