# Supplementary material for: Fish Snx27 promotes viral products by modulating the innate immune response and exosomal machinery
Source: J Virol. 2024 Nov 4;98(12):e00974-24. doi: 10.1128/jvi.00974-24 (PMC11650975; doi:10.1128/jvi.00974-24)
Supplement: Table S4 — The formation of the hydrogen bond residues and distances between EcSnx27 and RGNNV-CP. [file jvi.00974-24-s0005.docx]

**Supplementary Table 4** The formation of the hydrogen bond residues and distances between *EcSnx27* and RGNNV-CP.

| **Domains** | **SNX27** | **RGNNV-CP** | **Hydrogen bond length (Å)** |
| --- | --- | --- | --- |
| **PDZ** | K117 | A289 | 3.2 |
|  | R70 | D231 | 2.6、3.1 |
|  | Q68 | N230 | 2.5 |
|  | Q68 | D317 | 2.4 |
| **NA** | Y155 | R321 | 3.3 |
| **PX** | R250 | D317 | 3.0 |
|  | E254 | R276 | 2.4 |
|  | D256 | Q322 | 2.9 |
|  | D256 | R276 | 2.6 |
| **FERM** | T347 | D275 | 2.4、3.5 |
|  | N345 | Y267 | 2.0 |
|  | Q344 | Y267 | 3.3 |
|  | Q344 | V278 | 3.4 |
|  | K340 | V278 | 3.4 |
| **FERM-like** | E481 | R297 | 3.2 |
|  | E482 | R297 | 2.7 |
